# Dr. Charles Edward Hutt

**Published:** 1912-09-14

**Authors:** 


DR. CHARLES EDWARD HUTT has died at Tottenham at the age of forty-seven. Dr. Hutt was very well known and one of the hardest workers in the British Medical Association. He was chairman of the North Middlesex Division in 1905, and to his efforts is mainly due the present standing of this division. Dr. Hutt qualified from St. Bartholomew's Hospital in 1888 at the Conjoint Board. He was at one time resident medical officer at the Hertford Infirmary. Since 1896 he had been in practice in Tottenham, where he was universally held in the highest esteem.

